# P-1311. Infectious diseases trends in migrant populations at a safety-net community hospital in Chicago

**DOI:** 10.1093/ofid/ofae631.1492

**Published:** 2025-01-29

**Authors:** Mashaal Khan, Stephanie Vergara-Mojica, Alexandra Zodo, Mirza Ali, Alfredo J Mena Lora

**Affiliations:** Ross University School of Medicine, Chicago, Illinois; Ross University, Chicago, Illinois; Ross University, Chicago, Illinois; Saint Anthony Hospital, Chicago, Illinois; University of Illinois Chicago, Chicago, Illinois

## Abstract

**Background:**

Chicago's recent increase in migrant populations has placed new demands on public health systems, particularly in managing infectious diseases within community hospitals. This study examines the impact of migration on the incidence of infectious diseases in a community hospital's emergency department (ED). Understanding disease patterns in migrants can inform public health strategies and improve healthcare delivery.

**Figure 1.**
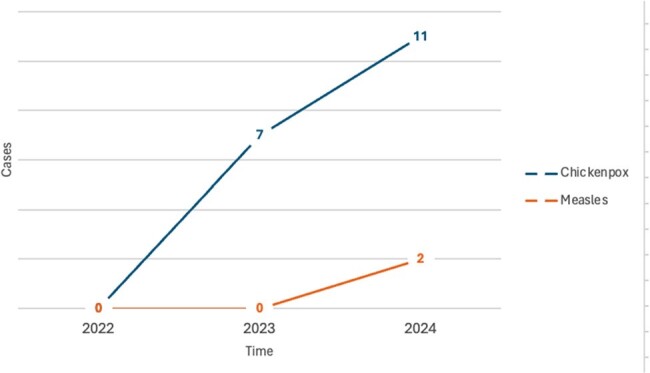
Chickenpox and measles cases per year

**Methods:**

We conducted a retrospective review of infection prevention records from a 151-bed Chicago safety-net community hospital was conducted, focusing on migrant patient visits and infection prevention needs between 2022 and 2024. Descriptive statistics were used to evaluate the changes over the period.

**Figure 2.**
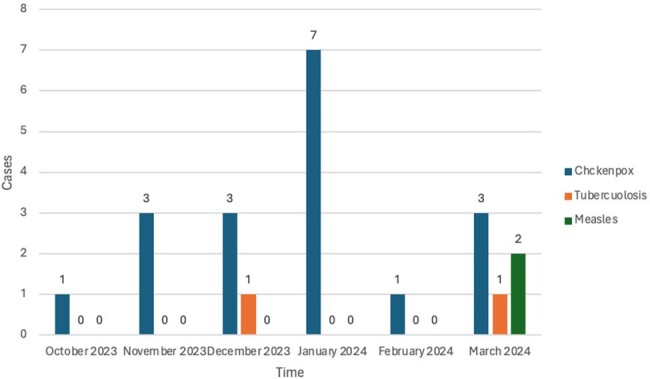
Infectious diseases cases in migrant populations per month

**Results:**

In 2022, 2023 and 2024, the reported cases of chickenpox rose from 0 to 5 and then 11, respectively. This represents a 450% increase in chickenpox cases from 2023 to 2024 (Figure 1). No measles cases were reported in the first two years, increasing to 2 cases in 2024. From October 2023 to March 2024, a total 18 chickenpox cases, 2 measles cases and two cases of tuberculosis were reported (Figure 2). Airborne precautions in the ED were needed for 22 cases.

**Conclusion:**

We report a notable increase in vaccine preventable conditions such as chickenpox and measles in a Chicago hospital associated with a migrant influx. Added needs for airborne precautions in the ED were required during this period. These trends highlight the need for public health measures, such as enhanced vaccination efforts and healthcare access for migrants, to prevent disease outbreaks and protect community health. Infection prevention readiness should involve clear protocols for identifying and isolating conditions requiring airborne precautions.

**Disclosures:**

**All Authors**: No reported disclosures

